# Factors impacting the benefits and pathogenicity of Th17 cells in the tumor microenvironment

**DOI:** 10.3389/fimmu.2023.1224269

**Published:** 2023-08-23

**Authors:** Jie Xing, Changfeng Man, Yingzhao Liu, Zhengdong Zhang, Huiyong Peng

**Affiliations:** ^1^ Department of Laboratory Medicine, The Affiliated People’s Hospital of Jiangsu University, Zhenjiang, China; ^2^ Department of Oncology, The Affiliated People’s Hospital of Jiangsu University, Zhenjiang, China; ^3^ Department of Endocrinology, The Affiliated People’s Hospital of Jiangsu University, Zhenjiang, China; ^4^ Department of Environmental Genomics, Jiangsu Key Laboratory of Cancer Biomarkers, Prevention and Treatment, Collaborative Innovation Center for Cancer Personalized Medicine, Nanjing Medical University, Nanjing, China; ^5^ Department of Genetic Toxicology, The Key Laboratory of Modern Toxicology of Ministry of Education, Center for Global Health, School of Public Health, Nanjing Medical University, Nanjing, China

**Keywords:** Th17 cells, tumor microenvironment, anti-/pro-tumor, molecular regulation, transdifferentiation

## Abstract

Tumor development is closely associated with a complex tumor microenvironment, which is composed of tumor cells, blood vessels, tumor stromal cells, infiltrating immune cells, and associated effector molecules. T helper type 17 (Th17) cells, which are a subset of CD4^+^ T cells and are renowned for their ability to combat bacterial and fungal infections and mediate inflammatory responses, exhibit context-dependent effector functions. Within the tumor microenvironment, different molecular signals regulate the proliferation, differentiation, metabolic reprogramming, and phenotypic conversion of Th17 cells. Consequently, Th17 cells exert dual effects on tumor progression and can promote or inhibit tumor growth. This review aimed to investigate the impact of various alterations in the tumor microenvironment on the antitumor and protumor effects of Th17 cells to provide valuable clues for the exploration of additional tumor immunotherapy strategies.

## Introduction

Malignant neoplasms represent a critical affliction in many nations and are characterized by high prevalence and mortality rates. Statistically, in over 100 countries, including China, the United States, the United Kingdom, Argentina, Spain, and South Korea, the proportion of deaths caused by cancer among noncommunicable diseases is 23.3%. As of 2020, the leading types of cancer in terms of incidence rates are breast cancer (11.7%), lung cancer (11.4%), prostate cancer (7.3%), nonmelanoma of the skin (6.2%), colon cancer (6.0%), and stomach cancer (5.6%) (1, 2). The increase in tumor incidence can be attributed to multifaceted pathogenesis, including the establishment of a tumor microenvironment that serves as a vital habitat for tumor sustenance (3–5). The tumor microenvironment is composed of diverse constituents, including neoplastic cells, vascular structures, stromal cells, infiltrating immunocytes, and assorted secreted effector molecules (6, 7). The inherent uniqueness of tumors provides novel therapeutic approaches for cancer treatment beyond established interventions such as surgical resection and chemotherapy. These approaches include tumor immunotherapy, such as chimeric antigen receptor T-cell therapy, cytokine therapy and miRNA drugs (8–10). However, neither T cell transfer therapy nor immune checkpoint therapy is universal, and they are only effective against a limited number of tumors (11, 12).

In order to address the limitations of these treatments and explore novel therapeutic approaches, extensive and in-depth research has been conducted on the intricate relationship between the tumor microenvironment and immune cells. This endeavor has even given rise to a new conceptual framework known as the ‘tumor immune microenvironment’, which is aimed at elucidating the role of immune cells and their effector molecules within the context of the tumor microenvironment (13). Immune cells within the tumor microenvironment play more than a singular role of promoting or inhibiting tumor progression. They display a contextual responsiveness, which is characterized as resistance or promotion based on dynamic changes in their surroundings. This effect transcends temporal and spatial limitations, meaning that these immune cells can manipulate tumors to vary degrees across different stages or locations within the same disease. In other words, these immune cells possess spatiotemporal adaptations (14). For instance, macrophages exhibit an antitumor M1 phenotype when exposed to high glucose and IFN-γ levels, whereas these cells acquire a protumor M2 phenotype in high lactate environments and in the presence of Colony-stimulating factor 1 (CSF1) (15–17). The polarization of neutrophils associated with tumors is influenced by the presence of transforming growth factor-β (TGF-β) in the local milieu (18, 19). Th17 cells follow a similar pattern, although they lack the clear functional categorization observed in the aforementioned cell types (20, 21).

Th17 cells are a type of CD4^+^ T cell that produces IL-17 and whose development is mainly regulated by transcription factors such as RORγt and STAT3, as well as various cytokines such as IL-6, IL-1β, IL-21, IL-23, and TGF-β. IL-6 and TGF-β induce RORγt transcription in CD4^+^ T cells, promoting the expression of IL-17 and IL-17F in Th17 cells. RORγt^+^ T cells act with IL-6 to upregulate IL-23R expression, facilitating the response to IL-23. This contributes to the maintenance, expansion, or further differentiation of Th17 cells. The combined signaling of TGF-β with IL-6/STAT3 and IL-21/STAT3 drives the differentiation of Th17 cells by promoting the expression of the transcription factors RORγt and RORα. STAT3 also directly binds and transactivates the promoters of IL-17 and IL-21, facilitating their expression (22–28).

Th17 cells have been shown to play an important role in various autoimmune diseases (29–31). In recent years, due to increasing research on tumors and inflammation, tumor microenvironments, and immune cells, the role of Th17 cells in tumors has been further explored. Through various studies on their role in tumors, the dual nature of Th17 cells in tumors cannot be ignored. Many scholars have suggested why Th17 cells promote or inhibit tumor growth and development, such as by promoting tumor angiogenesis to provide a rich blood supply to tumors, thereby promoting tumors, which occurs in melanoma, bladder cancer, hepatocellular carcinoma, and colorectal cancer (11, 20, 32). However, there is also evidence that Th17 cells can play an antitumor role by recruiting Cytotoxic T lymphocytes (CTLs) and Natural killer (NK) cells, such as in ovarian cancer and lung cancer (32). Most research has focuses on how downstream effectors of Th17 cells regulate tumor development. How upstream molecules affect Th17 cells in tumor microenvironments is still up for debate. Therefore, this article will focus on how the tumor microenvironment affects the dual function of Th17 cells.

## The biogenesis and functions of Th17 cells

After being stimulated by IL-6, IL-21, TGF-β, and other factors, naive CD4^+^ T cells are activated, leading to the differentiation of Th17 cells through the activation of STAT3 by RORγt (33, 34). These cells play a crucial role in host defense against pathogens and inflammatory responses (35, 36).Additionally, they are involved in the development of autoimmune diseases and cancer (37, 38). Th17 cells exert different functional effects depending on the environmental conditions and the type of cytokine stimulation they receive. TGF-β and IL-6 promote the generation of nonpathogenic Th17 cells from naive CD4^+^ T cells, mainly due to TGF-β mediated inhibition of T-bet expression. At this stage, Th17 cells can secrete IL-9 and IL-10, and IL-10 exerts antagonistic effects on Th17 cells pathogenicity during infection or inflammation (39, 40). Additionally, research has shown that TGF-β derived from Th17 cells play an important role in maintaining Th17 cells stability and blocking their pathogenic transformation by downregulating IL-12 and IL-27 expression (41). TGF-β can also inhibit the differentiation of pathogenic Th17 cells by suppressing the extracellular-signal-regulated kinase (ERK) pathway, whereas the TGF-β superfamily cytokine Activin-A can activate the ERK pathway through Activin-A receptor ALK4 to promote the differentiation of pathogenic Th17 cells (42). IL-23 and IL-1β can also stimulate Th17 cells differentiation, but since immature T cells do not express IL-1R and IL-23R, their effects mainly occur after Th17 cells differentiation begins (43, 44). Furthermore, IL-1β inhibits the production of IL-10, indicating that Th17 cells at this stage tend to promote disease development (45). Finally, IL-23 participates in Th17 cells pathogenicity (43). The transcription factors involved in the differentiation and functional regulation of Th17 cells are numerous. Among these, the most critical factors are STAT3 and RORγt. STAT3 is activated in response to stimulation by the JAK family kinases and requires subsequent activation of RORγt to drive Th17 cells differentiation (46). RORα shares gene binding sites with RORγt, and in the presence of the latter, it can cooperatively bind to RORE or DNA to enhance the inflammatory effects of Th17 cells (47). The presence of basic leucine zipper transcription factor ATF-like (BATF) promotes the expression of STAT3 while inhibiting IL-2-dependent STAT5, thereby inhibiting the competitive binding between STAT3 and STAT5. This disruption inhibits the differentiation of T cells towards Th1 cells and Treg cells subsets and facilitates the early differentiation of Th17 cells, which is associated with the expression of IL-17, IL-21, and IL-22 (48–50). Interferon regulatory factor 4 (IRF4) synergistically upregulates RORγt and downregulates FOXP3 in collaboration with STAT3, thereby promoting the differentiation of Th17 cells. The presence of IL-6 intensifies the aforementioned response and facilitates the expression of IL-17 and IL-21 (51, 52). The expression of aryl hydrocarbon receptor (AHR) is upregulated in Th17 cells, particularly in pathogenic Th17 cells, and the levels are sustained and stable. The upregulation of AHR is associated with the presence of TGF-β1 and IL-6, which facilitates the secretion of IL-17A and IL-22 by Th17 cells (53). The transcription factors T-bet and E26 transformation specific-1 (ETS-1) positively regulate Th1 cells and Th2 cells, respectively, and can inhibit Th17 cells differentiation. In the presence of IL-23, T-bet negatively modulates IL-17 secretion by Th17 cells. Moreover, ETS-1 negatively regulates Th17 cells by activating STAT5 in an IL-2-dependent manner (49, 54, 55). The transcription factor growth factor-independent 1 transcriptional repressor (GFI1), which is associated with the Th2 cells phenotype, act as a negative regulator of Th17 cells. IL-4 is required to inhibit the expression of RORγt and IL-17, but this inhibitory effect can be disrupted by TGF-β (56). Similarly, the transcription factor FOXP3, which is involved in Treg cells regulation, can suppress Th17 cells differentiation by inhibiting RORγt in the presence of TGF-β (57).To efficiently reach the site of inflammation, Th17 cells share transport receptors with Th1 cells, Th2 cells, and Treg cells. They share the receptors CCR4, CCR5, CCR6, CXCR3, and CXCR6 with Treg cells, while they share the receptors CCR2, CCR5, CCR7, CXCR3, and CXCR6 with Th1 cells. Th2 cells share the receptors CCR4 and CCR7 (58).This receptor sharing not only improves the migration of Th17 cells but can potentially define a distinct subgroup of Th17 cells. These Th17 cells express CCR6, produce CCL20, contribute to the recruitment of Th17 cells, are closely associate with IL-17 production, and participate in the inflammatory microenvironment associated with Th17 cells responses. These receptors may serve as pathogenic molecular markers of Th17 cells and are related to FOXP3^+^Th17 cells (31, 59).Notably, Th17 cells expressing CCR2 also express T-bet and RUNX family transcription factor 1 (RUNX1) and can secrete IFN-γ and GM-CSF, which are implicated in the pathogenicity of Th17 cells (60, 61).Th17 cells and neutrophils can mutually recruit each other. Neutrophils chemotactically attract Th17 cells through CCL2 and CCL20, while Th17 cells chemotactically attract neutrophils by producing and releasing CXCL8 (62).The effector molecules involved in the function of Th17 cells are IL-17A, IL-17F, IL-21, IL-22, and GM-CSF. Among these, IL-17A and IL-17F primarily contribute to host defense against bacteria and participate in mucosal defense. IL-17A can inhibit the pathogenicity of Th17 cells by inducing NF-κB activation and IL-24 production (63–65). IL-21 promotes Th17 cells expansion through self-amplification by stimulating RORγt and inhibits the expression of FOXP3 (66, 67) (68), while IL-22 is involved in tissue repair and resistance to pathogens (69). In addition to genetic factors, cellular metabolism influences the pathogenicity of Th17 cells. Th17 cells promote disease progression under conditions of active glycolysis and high tricarboxylic acid (TCA) activity, while protective Th17 cells seem to rely on fatty acid oxidation for energy (70). However, Wang et al. also discovered that when CD5L, which is a member of the scavenger receptor cysteine-rich superfamily, is inhibited by IL-23 in Th17 cells, it can alter lipid metabolism pathways and subsequently affect the downregulation of RORγt expression, leading to the generation of pathogenic Th17 cells (71). Furthermore, the active polyamine metabolism pathway promotes the generation of FOXP3^+^ Th17 cells and contributes to disease progression through the regulation of Jumonji domain-containing protein-3 (JMJD3) (70). Under different environmental conditions, Th17 cells exhibit varying functions based on the stimuli received. The complex tumor microenvironment is likely to cause a series of functional changes in Th17 cells. In the following section, this article will delve into various aspects of how the tumor microenvironment impacts the pathogenicity of Th17 cells (**Table 1**).

**Table 1:** Effectors that regulate Th17 cells differentiation, development, function, and pathogenicity.

	Molecules associated with Th17 cells differentiation and function	Mechanism of action	Relevance to pathogenicity	References
Cytokines	IL-21	Self-amplification promotes Th17 cells expansion and inhibits FOXP3 expression	A protective effect, possibly related to IRF4	(66, 67) (68),
	IL-6	Activates STAT3 in conjunction with TGF-β to regulate Th17 cells differentiation		(72)
	TGF-β	Activates the JAK -STAT3 pathway	By inhibiting T-bet, promoting IL-10 secretion, downregulating IL-12 and IL-27, and suppressing the ERK pathway, Th17 cells pathogenicity is disrupted	(39–41)
	IL-23	After the initiation of Th17 cells differentiation, further enhancement of Th17 cells differentiation occurs	Involved in the regulation of pathogenic Th17 cells	(43)
	IL-1β		Promotes Th17 cells pathogenicity by inhibiting IL-10	(45)
	IL-17A	Involved in host defense against bacteria and participates in mucosal defense	Activation of NF-κB and IL-24 serves to inhibit the pathogenicity of Th17 cells	(63, 64)
	IL-17F			
	GM-CSF	Secreted by Th17 cells, promotes further differentiation through IL-23 and IL-1β	Closely linked to pathogenic Th17 cells	(65)
Transcription factors	STAT3	Activates RORγt through the JAK -STAT3 pathway, crucial for early Th17 cells differentiation	Contributes to Th17 cells pathogenicity	(46, 73)
	RORγt and RORα	Key regulators of Th17 cells differentiation, stimulate IL-17 production		(33, 74)
	BATF	Facilitating STAT3 activation while inhibiting STAT5; inhibiting their competitive binding enhances early differentiation of Th17 cells. which is associated with the expression of IL-17, IL-21, and IL-22		(48–50)
	IRF4	Upregulates RORγt and downregulates FOXP3 in collaboration with STAT3, promoting the differentiation of Th17 cells. Stimulates secretion of IL-17 and IL-21		(51, 52)
	AHR	Influences expression of IL-17A and IL-22	Associate with pathogenic Th17 cells and sustained and stable levels	(53)
	NF-κB	Synergistically promotes Th17 cells differentiation via RORγt and RORα	Potentially involved in regulating pathogenic Th17 cells	(75, 76)
	T-bet	Suppresses Th17 cells differentiation by inhibiting RORγt	May inhibit Th17 cells pathogenicity	(77)
	ETS-1	Inhibits Th17 cells differentiation through STAT5 in an IL-2-dependent manner		(49, 54, 55)
	GFI1	Relies on IL-4 to inhibit the expression of RO Rγt and IL-17		(56)
	FXOP3	Antagonizes RORγt to inhibit Th17 cells differentiation	Promotes the conversion between Treg cells and Th17 cells, facilitates Th17 cells pathogenicity	(57)
Chemokine Receptors	CCR6	Recruits Th17 cells and is associated with FOXP3^+^Th17 cells	Participates in regulating pathogenic Th17 cells	(31, 59)
	CXCR3	Recruits Th17 cells		(78)
	CXCR6	Recruits Th17 cells	Involved in regulating pathogenic Th17 cells	(79)
	CCR2	Express T-bet and RUNX1 and can secrete IFN-γ and GM-CSF	Participates in regulating pathogenic Th17 cells	(57, 58)

## Metabolic alterations and Th17 cells

**Figure 1:**
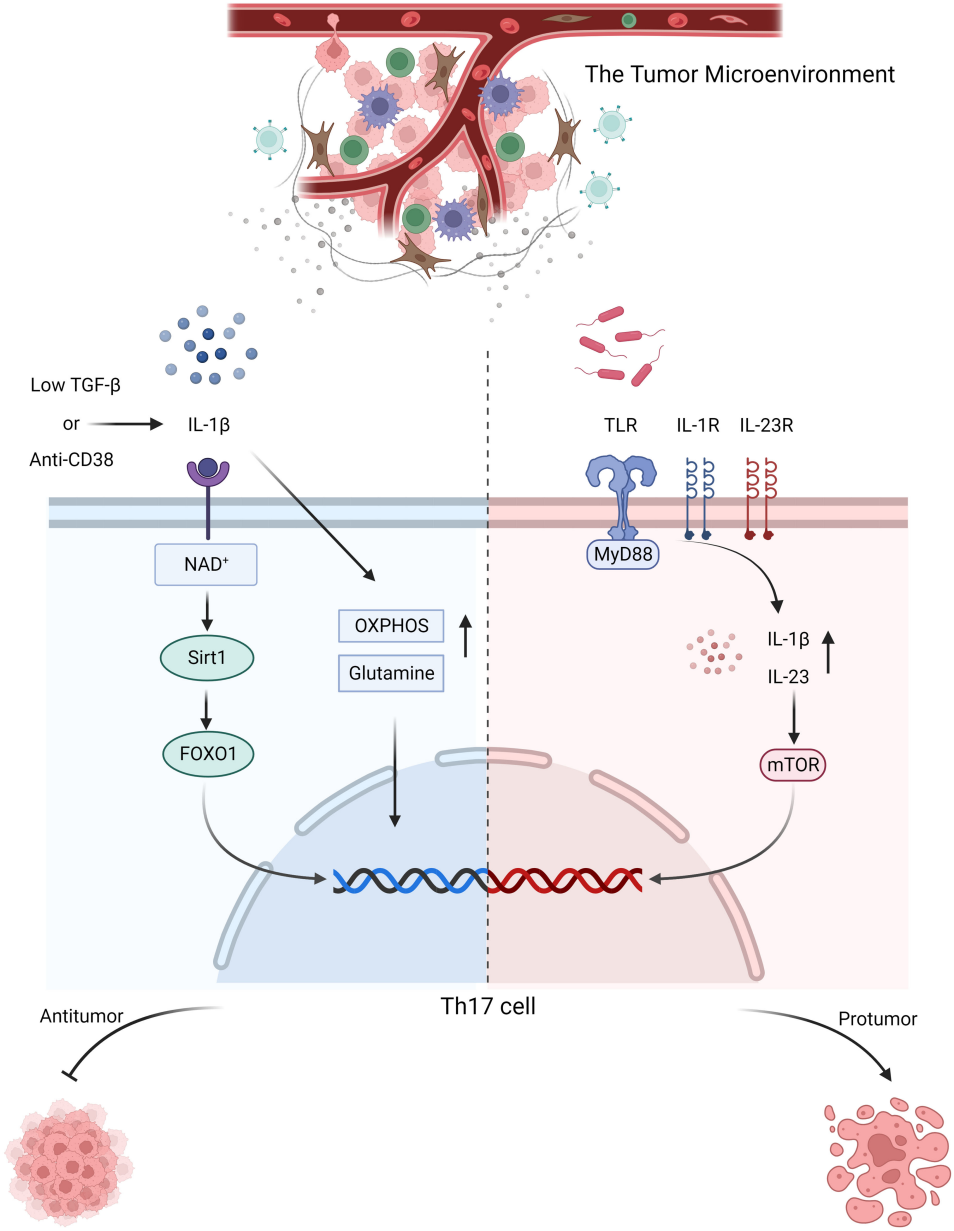
In response to stimulation with anti-CD38 or low-dose TGF-β combined with IL-1β, Th17 cells activate NAD^+^-histone deacetylase SIRT1-FOXO1 axis and undergo metabolic reprogramming, adopting a more active glutamine and OXPHOS metabolic pathway to exert their antitumor effects. In colorectal cancer, microbial signals emitted by commensal microorganisms are received by MyD88 through toll-like receptor, which sequentially activates IL-1R and IL-23R on the surface of CD4^+^ T cells, thereby inducing the production of IL-1β and IL-23. Consequently, mTOR expression is upregulated, promoting the proliferation and differentiation of Th17 cells, which in turn contribute to tumor promotion.

Tumor cells, tumor-infiltrating lymphocytes, and other immune or stromal cells within the tumor microenvironment compete for limited nutrients, and certain metabolic byproducts generated within the tumor microenvironment can inhibit antitumor immunity. Therefore, understanding the various metabolic conditions within the tumor microenvironment and their impact on Th17 cells is crucial for harnessing metabolic substances within the microenvironment to regulate tumors. In colorectal cancer, elevated levels of reactive oxygen species (ROS) disrupt glycolysis and PKM2-dependent phosphorylation of STAT3, thereby inhibiting the differentiation of Th17 cells. Activation of the PRAK-NRF2-ROS axis could inhibit ROS production and promote the differentiation of Th17 cells, leading to the induction of an antitumor effect (80). However, pathogenic Th17 cells are also present in colorectal cancer. The active glycolytic pathway is inhibited, thereby enhancing the inflammatory response and facilitating the disease progression. The origin of these Th17 cells is likely associated with the transdifferentiation of Treg cells (81). The active glycolytic environment plays a role in the antitumor effect of Th17 cells, but it falls short in sustaining their long-term antitumor efficacy. Studies have indicated that Th17 cells can secrete IL-17 and low levels of IFN-γ under glycolytic and oxidative phosphorylation (OXPHOS) metabolic conditions. In the tumor microenvironment, OXPHOS-mediated induction of BCL-XL and inhibition of BIM contribute to Th17 cells survival and prevent apoptosis. This fosters the stemness of Th17 cells and plays a crucial role in maintaining their antitumor capacity. Additionally, OXPHOS can enhance the use of glutamine by Th17 cells, promoting their differentiation (82). Wyatt et al. also supported this viewpoint in their investigation of antitumor Th17 cells. They found that the antitumor activity of Th17 cells was significantly enhanced by weak inducible T cell co-stimulator (ICOS) signaling or low TCR signaling. Additionally, these cells rely on glycolysis and oxidative phosphorylation, along with a memory cell phenotype (83). High levels of insulin-like growth factor 1 (IGF1) and insulin in obesity downregulate Glycogen synthase kinase 3 beta (GSK3B) activity by phosphorylating its serine residue, thereby altering the IL-17 signaling pathway, including upregulation of CXCL1, CCL20, and IL-6 expression. Melatonin can inhibit the upregulation of IL-17 by enhancing GSK3B activity. Preliminary validation has been conducted in human prostate cancer by Ge, D. et al. and there is evidence suggesting that IL-17 promotes prostate cancer progression (84, 85). This finding suggests that melatonin can exert its antitumor effects by inhibiting IL-17, but direct evidence is still required for confirmation. Apart from glucose and lipid metabolism, Filip-Psurska et al. constructed a mouse model of breast cancer and proposed that vitamin D and its metabolites could mediate the expression of osteopontin through VDR, thereby recruiting Th17 cells and promoting tumor progression. Osteopontin can serve as a tumor-associated inflammatory mediator, facilitating tumor metastasis, and its association with Th17 cells has been demonstrated in autoimmune diseases (86, 87). Considering the significant impact of metabolism on the plasticity of Th17 cells, the emerging trend in cancer treatment is the development of suitable antitumor Th17 cells for adoptive cell therapy (88). By constructing Th17/Th1 cells, which is a group of cells that possesses the stem cell characteristics of Th17 cells and the tumor-suppressive IFN-γ secretion abilities of Th1 cells, these composite cells exhibit a metabolic state characterized by suppressed glycolysis and enhanced glutamine breakdown and exert high expression of histone deacetylase sirtuin-1 (SIRT1) and Forkhead box O1 (FOXO1) under the conditions of a CD38 inhibitor and increased nicotinamide adenine dinucleotide^+^ (NAD^+^). Their antitumor effect is significantly enhanced compared to that of Th1 cells, as confirmed in mouse melanoma and prostate cancer. During the screening of composite cells, it was discovered that both IL-1β and TGF-β could stimulate the production of IFN-γ in composite cells for tumor suppression. However, IL-1β was more effective than TGF-β in this regard. This is because TGF-β induces the expression of CD73, which converts ATP to adenosine and inhibits the production of IFN-γ, thus counteracting some of the antitumor effect. Additionally, IL-1β is associated with more active glycolytic metabolism. Although IL-1β-induced Th17 cells have a favorable effect, they lack long-term stem cell properties. On the other hand, the presence of TGF-β can enhance their stem cell characteristics. Therefore, after adjusting the concentration of TGF-β, it was found that a low concentration of TGF-β in combination with IL-1β yields the optimal antitumor effect and stimulated Th17 cells (89–91) (**Figure 1**).

## Tumor antigens and Th17 cells

Apart from the metabolic microenvironment’s influence on Th17 cells, tumor-associated antigens can impact Th17 cells. This was demonstrated by van der Bruggen et al., who discovered that T cells could recognize the human melanoma antigen encoded by MAGE-1 (92). Tumor antigens have emerged as novel targets for antitumor immune therapy (93). However, the number of tumor antigens that can be recognized by T cells is limited, which is one of the limitations of immune checkpoint therapy (94). Jiao et al. suggested that the poor treatment efficacy of prostate cancer may be related to the number of tumor antigens and tumor-infiltrating T cells. Sufficient antigens are required to activate T cells to exert antitumor effects, but in reality, only a few antitumor T cells are activated, resulting in poor treatment effects (95). However, tumor antigens may not be scarce in number but are rather hidden by tumors. Therefore, the authors proposed the use of combination therapy to release these hidden antigens. The process of bone resorption that occurs in prostate cancer bone metastasis releases large amount of TGF-β and IL-6, which can activate Th17 cells. However, this process also reduces the activation of Th1 cells, which are the main antitumor cells. Although it is still unclear whether Th17 cells inhibit or support tumors in this context, blocking the appearance of Th17 cells can awaken the hidden antigens that activate Th1 cells, and combined with exogenous Th1 cells, should be effective in antitumor therapy.

## Microbial community dynamics and Th17 cells

The stimulation of Th17 cells differentiation by MyD88 can be divided into two parts. First, Th17 cells polarization is achieved through IL-1, followed by activation of the mTOR signaling pathway via IL-23, which further promoted Th17 cells differentiation. Research has indicated that the symbiotic microbiota in mouse models of colon cancer is recognized by Toll-like receptors, which stimulate MyD88, resulting in upregulated expression of IL-23 and promotion of IL-17 secretion by Th17 cells, thus facilitating tumor progression. This association may be linked to mTOR. In other words, microbial signals are received by MyD88 and subsequently activate IL-1R and IL-23R on the surface of CD4^+^ T cells. This activation leads to the production of IL-1β and IL-23, which, in turn, upregulate mTOR expression, facilitating the proliferation and differentiation of Th17 cells. These protumorigenic effects of Th17 cells can be inhibited by antibiotic therapy. Similarly, antibiotic treatment has been used on pancreatic cancer, although direct evidence is lacking, it is highly likely that the microbiota-mediated protumorigenic effects of Th17 cells on pancreatic cancer are also related to MyD88 (96–98) (**Figure 1**). The same tumorigenic effect was also confirmed in a study by Dmitrieva-Posocco, Oxana et al. The increase in tumor-promoting gut bacteria in the mouse model of colorectal cancer induced by the APC gene upregulated IL-1R1 expression on T cells, which in turn stimulated the production of IL-17 and IL-22, thereby promoting tumor progression (99). However, there is also evidence suggesting that Th17 cells exert antitumor effects on murine colorectal cancer. The presence of the gut microbiota has been shown to inhibit IL-6 and IL-1, thereby suppressing the differentiation of Th17 cells and promoting tumor progression. This finding contradicts the findings of Wang et al. and Dmitrieva-Posocco, Oxana et al. The author’s viewpoint attributes this contradiction to differences in the methods of constructing colorectal cancer models, animal models, and the cytokine milieu within the tumor microenvironment. It also underscores the reciprocal interaction between the tumor microenvironment and Th17 cells. Therefore, when studying the role of Th17 cells in tumors, it is essential to consider the composition of various components within the tumor microenvironment and their impact on the proliferation and differentiation of Th17 cells (100). The overexpression of miR-149-3p has been shown to ameliorate Th17 cells infiltration and colitis induced by Enterotoxigenic Bacteroidesfragilis, thereby inhibiting the progression to colorectal cancer. This finding not only suggests the impact of the microbe-associated inflammatory response mediated by Th17 cells on cancer progression but also highlights the significant role of Th17 cells in the transition from precancerous lesions (colitis) to colorectal cancer (101). Similarly, the presence of Th17 cells has been observed in the progression from colorectal adenoma to colorectal cancer; however, it remains unclear whether microbes are involved in this process (102). The presence of *Helicobacter pylori* in gastric cancer also promotes the development of Th17 cells, contributing to a tumorigenic effect (103). These findings indicate that the microbiota contribute to the regulation of Th17 cells in the tumor microenvironment, and to a large extent, they are associated with tumor-promoting effects.

## The signaling molecules contributing to Th17 cells in the tumor microenvironment

Whether it is the tumor metabolic microenvironment, tumor antigens, or the microbiota, their impact on Th17 cells pathogenicity relies on signaling pathways and the involvement of effector molecules. The study of tumors and Th17 cells have revealed numerous intersections between the two, indicating that tumors attract Th17 cells while creating a suitable microenvironment for themselves. In their investigation of different stages of breast cancer, Avalos-Navarro et al. discovered that Macrophage migration inhibitory factor (MIF) promoted the production of TGF-β, IL-6, and IL-1 while also participating in the activation of signaling pathways such as NF-kB, STAT3, PI3K/AKT, and MAPK (ERK1/2), thus contributing to Th17 cells generation. Moreover, they demonstrated a positive correlation between MIF and tumor progression mediated by Th17 cells (104). In their investigations on breast cancer and lung cancer in humans and mice, Voigt et al. revealed that IL-22 facilitated tumor progression, and Th17 cells were identified as one of the primary cellular sources. NOD-like receptor family pyrin domain containing 3 (NLRP3) inflammasome activation within the tumor microenvironment can induce the activation of IL-1β, which subsequently activates RORγt to promote the secretion of IL-22 by Th17 cells, thereby promoting disease advancement (105). This finding indicates that tumors exploit signaling pathways that are advantageous to themselves, substituting the normal signals sent by the body to Th17 cells and thereby using Th17 cells to promote tumor growth. The study by Fabre et al. in skin cancer supports this possibility, as overexpression of STAT3 promoted tumor progression by stimulating the angiogenic effects of IL-17^+^ Th17 cells (106). Additionally, research by Xu, Z. S. revealed that Th17 cells facilitated the progression from colitis to colorectal cancer. Family with sequence similarity 64, member A (FAM64A), which is a positive regulator of STAT3, enhances IL-6-mediated STAT3 activation, promoting Th17 cells differentiation and facilitating inflammation-induced carcinogenesis (107). The expression of β-catenin in T cells is elevated in colorectal polyps, leading to the polarization of T cells towards Th17 cells through the activation of RORγt. This process promotes the occurrence of intestinal inflammation and the progression to tumor formation. Additionally, stable expression of β-catenin downregulates FOXP3 expression in Treg cells while upregulating RORγt, facilitating the conversion of Treg cells and exacerbating inflammation to promote disease progression (108). Apart from transcription factors, the presence of certain cytokines also regulates the function of Th17 cells. The study by Fabre et al. highlighted the similar roles of tumor-associated cytokines, such as IL-6. Skin cancer cells are characterized by their ability to secrete IL-6. Blocking IL-6 leads to tumor regression, as the protumor activity of IL-17 secreted by Th17 cells is regulated by IL-6 and STAT3 (109). Similarly, within the colorectal cancer microenvironment, DC cells with liver kinase B1 (LKB1) deficiency can promote tumor progression by activating the IL-6-STAT3 axis and facilitating Th17 cells differentiation (110). In KRAS-mutant lung cancer, inhibiting MEK increases the expression of TGF-β, IL-6, and IL-23, promoting the differentiation of Th17 cells and the secretion of IL-17 and IL-22, thereby enhancing tumor resistance, invasiveness, and metastasis. The presence of IL-17 increases tumor resistance by upregulating CD38, while IL-22 is associated with EMT, strengthening tumor invasiveness and metastasis. Targeted therapies can provide more effective control over tumor progression (111). Th17 cells also showed antitumor effects. Muranski et al. established a novel mouse model of melanoma called TRP-1 TCR transgenic mice. They observed that Th17 cells and Th1 cells exerted antitumor effects, and Th17 cells demonstrated superior efficacy compared to Th1 cells. This could be attributed to the increased secretion of IFN-γ and the sustained presence of Th17 cells. This aligns with the aforementioned notion of antitumor Th17 cells in the context of the microbiota and metabolism. These perspectives delineate some of the defining characteristics of antitumor Th17 cells: their persistence, stemness properties and capacity to secrete IFN-γ. Additionally, it reaffirms the influence of the tumor microenvironment on Th17 cells (112). Moreover, this subset of Th17 cells has already been defined. They exhibit abundant expression of BCL2 and BCL-XL, increased levels of stem cell-related genes, elevated expression of cell cycle proteins, and reduced CDK inhibitors. These cells also exhibit upregulated Wnt/β-catenin-associated gene expression and inhibit apoptosis through the HIF/Notch/BCL2 axis. Furthermore, they can induce the production of CXCL9 and CXCL10 by tumors to recruit Th1 cells for antitumor responses (113, 114). Knochelmann et al. further validated novel characteristics of antitumor Th17 cells using the same mouse model. The antitumor efficacy of Th17 cells was optimal on the fourth day of *in vitro* expansion, and was characterized by a memory cell phenotype (CD44^hi^ CD62L^lo^). These cells exhibited high levels of CD25, ICOS, and OX40 expression. The presence of IL-6 further contributed to the sustained and durable antitumor capacity of Th17 cells (115). Su et al. proposed that within the tumor microenvironment of melanoma (as well as ovarian cancer, breast cancer, and colon cancer), tumor cells and tumor-associated fibroblasts secrete monocyte chemoattractant protein-1 (MCP-1) and RANTES, which mediate the migration of Th17 cells into the tumor microenvironment. Tumor cells and stromal cells further promote the generation and expansion of Th17 cells by secreting IL-1, IL-6, IL-23, and TGF-β. Inflammatory signals induced by TLRs and Nod2 also contribute to the proliferation and generation of Th17 cells. Initially, these factors facilitate the proliferation of Th17 cells mediated by tumor cells and tumor-associated fibroblasts and subsequently enhance the recruitment of Th17 cells by upregulating MCP-1 and regulated upon activation normal T cell expressed and secreted factor (RANTES) levels. Finally, these signals promote the recruitment and differentiation of Th17 cells by dendritic cells, monocytes, PBMCs, and other immune cells. The aforementioned factors that regulate Th17 cells have been shown to promote disease progression *in vitro*, but further validation is required *in vivo (* 116). A similar mechanism of Th17 cells recruitment can be observed in malignant pleural effusion, where these cells also exert antitumor effects. Tumor cell-secreted CCL20 and CCL22 in the pleural effusion act on the corresponding receptors CCR4 and CCR6 on Th17 cells and recruit them (117). Additionally, in invasive bladder cancer, the recruitment of Th17 cells through chemokines is involved in exerting antitumor effects (118). These findings suggest that directly recruited Th17 cells can play an antitumor role within the tumor microenvironment. However, there are exceptions, as demonstrated in non-small cell lung cancer, in which CCL20 promotes tumor progression by recruiting Th17 cells (119). Apart from the effects of cytokines secreted by tumors themselves, immune cells in the tumor microenvironment also affect Th17 cells. Research by Li et al. has shown that the level of IL-6 was increased in tertiary lymphoid structures adjacent to hepatocellular carcinoma, which promoted the differentiation of Th17 cells and provided an active antitumor immune microenvironment. Moreover, the expression of CCR7 is high in peritumoral tissue, which may help stabilize Th17 cells in the tumor microenvironment (120). Furthermore, inhibiting secretion of immunosuppressive molecule IL-10 by B cells can also enhance the protumor effect of IL-17^+^ Th17 cells (121). Dendritic cells secrete IL-6, TGF-β, and IL-23 to induce polarization of Th17 cells (122).

## The transdifferentiation of Th17 cells and tumor microenvironment

**Figure 2:**
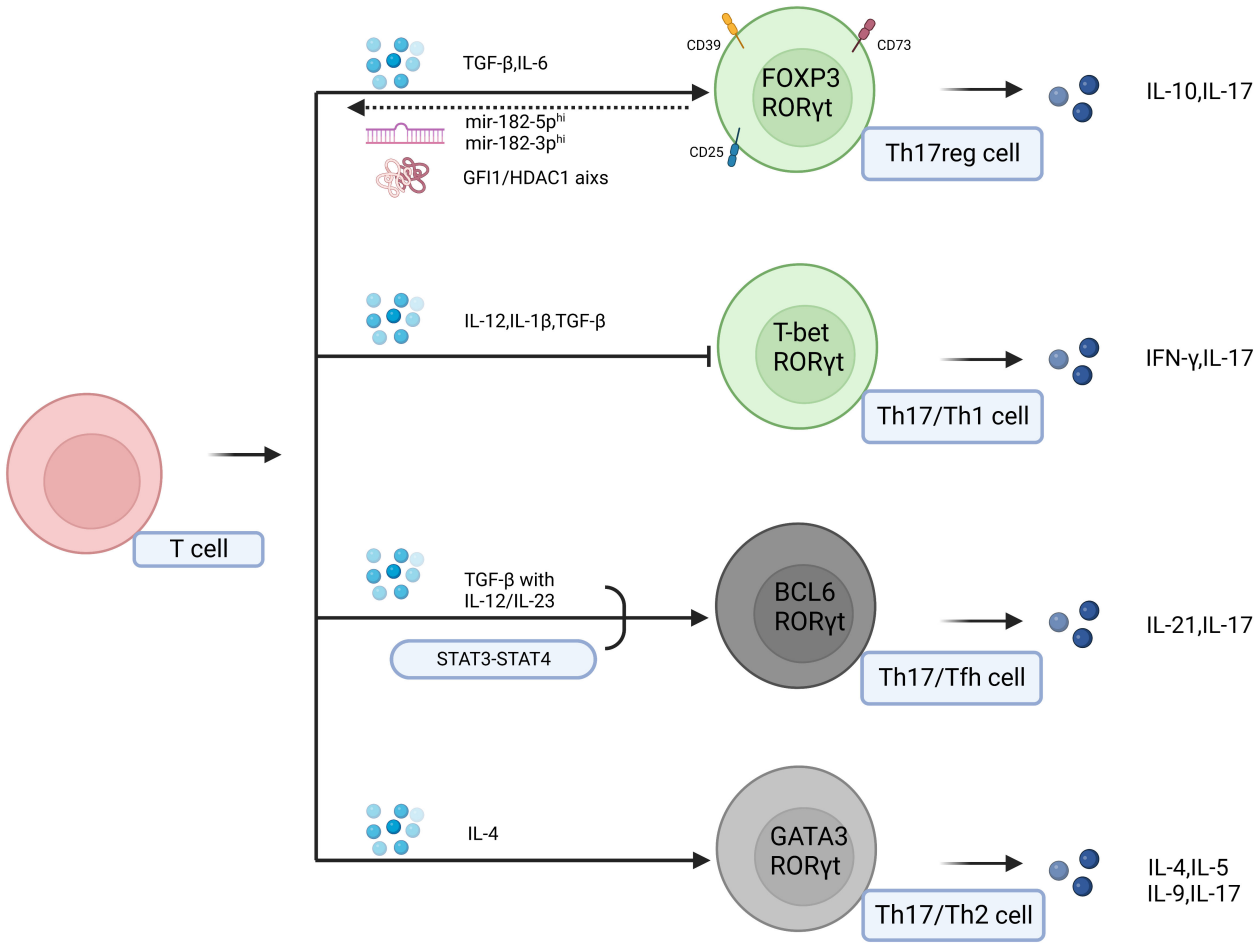
Effector molecules in the tumor microenvironment can induce the transdifferentiation of Th17 cells into Tfh cells, Th2 cells, Treg cells, and Th1 cells. In this context, Th17 cells can exhibit the characteristics and functions of Th17 cells as well as those of the transdifferentiated cells. The two populations of grey cells represent undiscovered transdifferentiation types within the tumor.

In addition to directly impacting Th17 cells to exert their antitumor effects, effector molecules in the tumor microenvironment induce the conversion of Th17 cells into Tfh cells, Th2 cells, Treg cells, and Th1 cells. In this context, Th17 cells can simultaneously possess the characteristics and functions of Th17 cells and converted cells (32, 123). This process of lineage conversion from one cell type to another is referred to as transdifferentiation (124). TGF-β can influence the reciprocal conversion between Th17 cells and Treg cells, leading to the coexpression of FOXP3 and RORγt in Treg cells and Th17 cells, which acquire dual functional capacities. The presence of AHR can also facilitate this conversion (125, 126). In breast cancer, TGF-β, PGE2 and blocking glycolysis facilitate the conversion of Th17 cells to Treg cells. These cells exhibit the phenotype of IL-17A^+^ FOXP3^+^ and possess the characteristics of Th17 cells, such as CD25 and CCR4 expression, as well as characteristics of Treg cells, such as CD161 and CD49d expression, thus promoting disease progression (127). Similarly, Roy et al. observed this phenomenon in breast cancer and proposed a Th17reg cells subset characterized by high expression of CD39 and CD73. The key mediator of this conversion is believed to be the GFI1/HDAC1 axis. Soheilifar et al. also identified a population of IL-17-secreting Treg cells whose differentiation into IL-17-secreting Treg cells was promoted by the overexpression of mir-182-5p and mir-182-3p. Marion Thibaudin discovered a subset of Th17 cells with high CD25 expression that accumulates in human breast cancer. Notably, these cell types also express CD39 and CD73, suggesting that the three aforementioned subsets are likely Th17reg cells (128–130). CD73 and CD39 have been shown to be signaling molecules that can reshape the immunosuppressive tumor microenvironment. These factors inhibit effector immune cells and activate immunoregulatory cells by dephosphorylating extracellular ATP to generate adenosine. These factors are express by not only Treg cells, but they are also expressed on various cell types within the tumor microenvironment. The expression of CD73 and CD39 on Th17 cells may be related to IL-6-STAT3 and TGF-β-GFI1, and GFI1 exerts inhibitory effects on the differentiation of Th17 cells and the expression of nucleases in Th17 cells. TGF-β can disrupt of these inhibitory mechanisms. IL-6, which is unrelated to GFI1, positively regulates the expression of CD73 and CD39 through STAT3 (131).This strongly indicates that the conversion of Th17 cells to Treg cells expressing CD73 and CD39 promotes tumor progression (132). The phenotypic conversion between Th17 cells and Treg cells in gastric cancer may arise from interactions among mesenchymal stem cells (MSCs), tumor-associated fibroblasts (PCCs), and gastric cancer stem cells (CSCs). The interplay between MSCs and CSCs prompts the secretion of TGF-β by MSCs, leading to the Th17 cells-to-Treg cells conversion, which is regulated by Notch signaling. In pancreatic cancer, the presence of Th17/Th1 cells phenotype has been identified. RIP1i-mediated macrophage reprogramming leads to the phenotypic reshaping of CD4^+^ T cells, resulting in high co-expression of IFN-γ, IL-17, T-bet, and RORγt. These cells lose the protumor capabilities associated with IL-17^+^ Th17 cells and acquire the antitumor capabilities of IFN-γ-producing Th1 cells, thus representing Th17.1 cells (133). In fact, the construction of Th17.1 cells has long been pursued for tumor therapy, as mentioned previously. These cells possess the characteristics described earlier, including a memory phenotype and the expression of IL-17 and IFN-γ. Furthermore, Muranski, P. et al. proposed that this cell population also expresses Tcf7 and β-catenin, providing evidence for the memory phenotype of Th17.1 cells and suggesting their superior antitumor capabilities compared to Th1 cells (134).In asthma, there is evidence of the conversion of Th17 cells into Th2 cells (135). Schmitt’s research indicates that in an inflammatory environment associated with autoimmune diseases, Th17 cells can acquire phenotypic characteristics of Tfh cells under the influence of TGF-β and other cytokines (136). This finding suggests the occurrence of transdifferentiation. However, this particular differentiation type has not yet been identified in tumors (**Figure 2**). In summary, the heterogeneity of Th17 cells is likely one of the reasons for their dual roles in tumor regulation (**Table 2**).

**Table 2:** Tumor information and the impact of the tumor microenvironment on Th17 cells.

Cancer	Incidence rate (%) *	Mortality rate (%) *	Effects of the tumor microenvironment on Th17 cells	
			Protumor	Antitumor
Breast	11.7	6.9	1, Vitamin D and its metabolites can mediate the expression of osteopontin through VDR to recruit Th17 cells (86, 87) 2, MIF promotes the production of TGF-β, IL-6, and IL-1 to promote the generation and expansion of Th17 cells (104) 3, NLRP3 inflammasome activation within the tumor microenvironment can induce the activation of IL-1β, which subsequently activates RORγt to promote the secretion of IL-22 by Th17 cells (105). 4, MCP-1 and RANTES recruit Th17 cells; IL-1, IL-6, IL-23, and TGF-β promote the generation and expansion of Th17 cells. Inflammatory signals induced by TLRs and Nod2 contribute to the proliferation and generation of Th17 cells (116) 5, TGF-β, PGE2 and blocking glycolysis facilitate the conversion of Th17 cells to Treg cells (127)	
Lung	11.4	18	1、 1, NLRP3 inflammasome activation within the tumor microenvironment can induce the activation of IL-1β, which subsequently activates RORγt to promote the secretion of IL-22 by Th17 cells (105). 2、 2, TGF-β, IL-6, and IL-23, promoting the differentiation of Th17 cells and secretion of IL-17 and IL-22 (111) 3, CCL20 recruits Th17 cells (119).	
Prostate	7.3	3.8	1, High levels of IGF1 and insulin in obesity downregulate GSK3B activity by phosphorylating its serine residue, upregulation CXCL1, CCL20, and IL-6 expression (84) 1、 2, Tumor antigens deficiency (95)	1, Th17/Th1 cells were induced by low concentrations of TGF-β in combination with IL-1β or a CD38 inhibitor to increase NAD+ (89–91)
Colon	6.0	5.8	1, Microbial signals are received by MyD88, which subsequently activates IL-1R and IL-23R on the surface of CD4^+^ T cells. This activation leads to the production of IL-1β and IL-23, which, upregulate mTOR expression, facilitating the proliferation and differentiation of Th17 cells (96–98) 2、MCP-1 and RANTES recruiting Th17 cells, IL-1, IL-6, IL-23, and TGF-β promote the generation and expansion of Th17 cells, Inflammatory signals induced by TLRs and Nod2 also contribute to the proliferation and generation of Th17 cells (116) 3、MondoA-TXNIP axis, promotes the polarization of Th17 cells and the production of IL-17A (81)	1, Activation of the PRAK-NRF2-ROS axis could inhibit ROS and promote the differentiation of Th17 cells (80)
Rectum	3.8	3.4	Same as in colon cancer	Same as in colon cancer
Melanoma	1.7	0.6	1、 1, The IL-6-STAT3 axis facilitate Th17 cells differentiation (110) 2、MCP-1 and RANTES recruit Th17 cells; IL-1, IL-6, IL-23, and TGF-β promote the generation and expansion of Th17 cells. Inflammatory signals induced by TLRs and Nod2 contribute to the proliferation and generation of Th17 cells (116)	1, Th17/Th1 cells were induced by low concentrations of TGF-β in combination with IL-1β or a CD38 inhibitor to increased NAD^+^ (89–91)
Pancreatic	2.6	4.7	1, Microbial signals are received by MyD88, which subsequently activates IL-1R and IL-23R on the surface of CD4^+^ T cells. This activation leads to the production of IL-1β and IL-23, which, upregulates mTOR expression, facilitating the proliferation and differentiation of Th17 cells (96–98)	1, RIP1i-mediated macrophage reprogramming leads to the phenotypic reshaping of CD4^+^ T cells, resulting in high coexpression of IFN-γ, IL-17, T-bet, and RORγt (133)

*Citation reference number (2).

## Discussion

Hanahan summarized eight fundamental hallmarks of tumors (3, 137). These eight hallmarks have resulted in a more structured study of tumor development. For example, IL-17 secretion can induce epithelial-mesenchymal transition (EMT) in lung cancer cells, promoting their migration and metastatic dissemination, which corresponds to the activation of invasion and metastasis (138). Additionally, IL-17 can promote tumor angiogenesis by coordinating with tumor fibroblasts to induce various angiogenic factors, including vascular endothelial growth factor, prostaglandin E2, and nitric oxide. corresponding to the tumor-induced vascular system (139, 140). Furthermore, apart from tumor promotion, IL-17 exhibits antitumor characteristics that correspond to the aforementioned features, such as indirectly attracting CD8^+^ T cells and NK cells to eliminate tumor cells (141). Th17 cells are involved in various stages of tumor development and exert antitumor and protumor effects. Therefore, the factors governing these divergent regulatory functions of Th17 cells must be considered, which constitutes the focal point of this review.

Th17 cells are inflammatory cells that plays a significant role in the context of inflammation and tumors. Exploring the signaling pathways shared among Th17 cells, tumors, and inflammation reveals an overlapping network of molecules and cytokines (142). Remarkably, Th17 cells tend to exert tumor-promoting effects, which are often mediated by overlapping signaling molecules or cytokines. This is because Th17 cells lack precise recognition capabilities and instead rely on the environment. Leveraging this understanding, we can strategically reshape the tumor microenvironment to enhance the antitumor capacity of Th17 cells. For instance, anti-PD-1 therapy upregulates the solute carrier family 11 member 1 (SLC11A1) gene in the tumor microenvironment, promoting the differentiation of Th17 cells. The levels of Th17 cells are significantly associated with improved overall survival (OS), suggesting that Th17 cells may serve as prognostic indicators for anti-PD-1 therapy (97, 143). Additionally, the microbiome’s involvement in intestinal tumors coordinates with the sustained presence of chronic inflammation, thus promoting tumor development by inducing the differentiation of Th17 cells (144). In fact, there are therapeutic approaches targeting these pathways in tumor therapy. For instance, modulation signaling pathways by manipulation of noncoding RNA expression, such as through overexpression or knockdown, can impact the function of Th17 cells and other immune cell subsets. Additionally, antibiotic treatment targets microbial infections can also combat tumors (101). However, these findings do not fully elucidate the additional aspects of the antitumor effect mediated by Th17 cells. In addition to inflammation, the plasticity of Th17 cells themselves may play a role in regulating tumor behavior (123, 145).There may be two distinct states of Th17 cells: classical Th17 cells and transdifferentiated Th17 cells, which are characterized by different phenotype traits such as Th17reg cells and Th1/Th17 cells. The existence of these diverse forms may be associated with the presence of TGF-β. Currently, chemokine recruitment of Th17 cells primarily involves classical Th17 cells, which subsequently undergo transformation in various directions in response to interactions with the microenvironment through processing and modification. It has been shown that the transdifferentiation of Th17 cells to Th1 cells and Th17 cells to Treg cells are reversible processes (146, 147). Additionally, metabolically, transdifferentiated Th17 cells differ from classical Th17 cells. For example, Th17reg cells may require lipid metabolism for activation, similar to Treg cells (148). However, clear evidence to support this notion is currently lacking. If proven, targeting cellular metabolic pathways to alter the transdifferentiation of immune cells could become a novel approach for tumor therapy. In summary, the assessment of the role of Th17 cells in the tumor microenvironment may require comprehensive consideration of the following factors: 1)cellular metabolism, including glycolysis and oxidative phosphorylation; 2) the level of signaling factors such as TGF-β, IL-6, and IL-23 in the tumor microenvironment, as well as the expression of STAT3, T-bet, FOXP3, IL-17, IL-22, IFN-γ, CD39, CD73 and chemotactic factors with cellular specificity in Th17 cells; and 3) the presence of a memory phenotype or long-lived characteristics in Th17 cells, as indicated by the expression of CD44^hi^, CD62L^lo^, and the involvement of stem cell-related genes such as Wnt/β-catenin and Tcf7. In conclusion, considering these factors comprehensively will facilitate more precise characterization of the effector functions of Th17 cells in tumors and enable more effective antitumor therapies.

## Author contributions

JX, and CM drafted the manuscript and drawn the tables and figures. ZZ conducted the project guidance. YL and HP designed the study and revised the manuscript. All authors contributed to the article and approved the submitted version.
